# Efficacy and safety of dasotraline in adults with binge-eating disorder: a randomized, placebo-controlled, fixed-dose clinical trial

**DOI:** 10.1017/S1092852920001406

**Published:** 2021-10

**Authors:** Carlos M. Grilo, Susan L. McElroy, James I. Hudson, Joyce Tsai, Bradford Navia, Robert Goldman, Ling Deng, Justine Kent, Antony Loebel

**Affiliations:** 1 Department of Psychiatry, Yale School of Medicine, New Haven, Connecticut, USA; 2 Lindner Center of HOPE, Mason, Ohio, USA; 3 Department of Psychiatry & Behavioral Neuroscience University of Cincinnati College of Medicine, Cincinnati, Ohio, USA; 4 Biological Psychiatry Laboratory McLean Hospital, Belmont, Massachusetts, USA; 5 Department of Psychiatry, Harvard Medical School, Boston, Massachusetts, USA; 6 Global Clinical Research, Sunovion Pharmaceuticals Inc., Marlborough, Massachusetts, USA; 7 Biostatistics, Sunovion Pharmaceuticals Inc., Marlborough, Massachusetts, USA; 8 Sunovion Pharmaceuticals Inc., Marlborough, Massachusetts, USA

**Keywords:** Binge-Eating Disorder, dasotraline, dopamine transporter, inhibitor, norepinephrine transporter, randomized controlled trial, obesity, weight loss

## Abstract

**Objective:**

The aim of this fixed-dose study was to evaluate the efficacy and safety of dasotraline in the treatment of patients with binge-eating disorder (BED).

**Methods:**

Patients meeting Diagnostic and Statistical Manual of Mental Disorders, Fifth Edition criteria for BED were randomized to 12 weeks of double-blind treatment with fixed doses of dasotraline (4 and 6 mg/d), or placebo. The primary efficacy endpoint was change in number of binge-eating (BE) days per week at week 12. Secondary efficacy endpoints included week 12 change on the BE CGI-Severity Scale (BE-CGI-S) and the Yale-Brown Obsessive–Compulsive Scale Modified for BE (YBOCS-BE).

**Results:**

At week 12, treatment with dasotraline was associated with significant improvement in number of BE days per week on the dose of 6 mg/d (N = 162) vs placebo (N = 162; −3.47 vs −2.92; *P* = .0045), but not 4 mg/d (N = 161; −3.21). Improvement vs placebo was observed for dasotraline 6 and 4 mg/d, respectively, on the BE-CGI-S (effect size [ES]: 0.37 and 0.27) and on the YBOCS-BE total score (ES: 0.43 and 0.29). The most common adverse events on dasotraline were insomnia, dry mouth, headache, decreased appetite, nausea, and anxiety. Changes in blood pressure and pulse were minimal.

**Conclusion:**

Treatment with dasotraline 6 mg/d (but not 4 mg/d) was associated with significantly greater reduction in BE days per week. Both doses of dasotraline were generally safe and well-tolerated and resulted in global improvement on the BE-CGI-S, as well as improvement in BE related obsessional thoughts and compulsive behaviors on the YBOCS-BE. These results confirm the findings of a previous flexible dose study.

## Introduction

Binge-eating disorder (BED) is the most common eating-disorder diagnosis, with an estimated lifetime prevalence of approximately 2.8% in women and 1.0% in men.^1^ ^–^^4^ BED is characterized by recurrent episodes of excessive food intake accompanied by a sense of loss of control during the over-eating, marked distress, and feelings of shame or guilt; however, unlike bulimia nervosa, patients with BED do not typically engage in regular weight-compensatory behaviors such as self-induced vomiting or use of laxatives, diuretics, or enemas.^5^

BED typically has an onset in early adulthood, a chronic course, and is associated with a high degree of comorbidity with other psychiatric disorders, most notably mood disorders (particularly major depressive disorder), anxiety disorders, and alcohol abuse/dependence.^1^ ^,^^2^ ^,^^6^ Medical comorbidity is also common in both epidemiologic and treatment-seeking BED populations, including obesity, hypertension, type 2 diabetes, and chronic pain conditions.^1^ ^,^^2^ ^,^^6^ ^–^^10^ Obesity (body mass index [BMI] ≥30 kg/m^2^) occurs in 30% to 50% of identified cases of BED, with higher rates observed in individuals with longer duration of illness.^2^ ^,^^11^ ^,^^12^

Despite the high prevalence and chronicity of BED, and high rates of psychiatric and medical comorbidity, the majority of individuals never receive treatment specifically for BED.^1^ ^,^^2^ ^,^^13^ Evidence for efficacy in the treatment of BED has been reported for various classes of medication, including selective serotonin reuptake inhibitors, anticonvulsants (topiramate and zonisamide), and stimulant medications (eg, lisdexamfetamine dimesylate, the only drug currently approved by the FDA).^14^ ^-^^19^ A considerable body of evidence also supports use of cognitive-behavioral and interpersonal therapies for the treatment of BED.^20^ ^,^^21^

Dasotraline is an inhibitor of dopamine and norepinephrine transporters, with a pharmacokinetic profile characterized by slow absorption and a long elimination half-life (t½, 47-77 hours). This results in stable plasma concentrations over 24 hours and permits once-daily dosing.^22^ ^,^^23^ The potential therapeutic benefit of dasotraline in BED, suggested by its pharmacologic profile, has been confirmed in a rat model of binge-like eating, where dasotraline showed a significant dose-related reduction in binge-like consumption of chocolate, with a smaller reduction in consumption of chow.^24^ In a prior randomized, double-blind, placebo-controlled trial in adults with BED, treatment with flexible doses of dasotraline, 4 to 8 mg/d was associated with significant week 12 reduction in binge-eating (BE) days per week (*P* < 0.001; effect size [ES]: 0.74).^25^ The aim of the current study was to replicate these findings by evaluating the efficacy and safety of two fixed doses of dasotraline (4 and 6 mg/d) in adults with BED.

## Methods

Eligibility requirements included that adults (18-55 years) met Diagnostic and Statistical Manual of Mental Disorders, Fifth Edition criteria for BED,^5^ confirmed using the Eating Disorders Module H of the Structured Clinical Interview for DSM disorders^26^ and relevant behavioral items on the Eating Disorder Examination Questionnaire (EDE-Q; eg, presence of BE and absence of weight-compensatory behaviors).^27^ ^-^^29^ Additionally, moderate-to-severe BED was required, based on a history of ≥2 BE days per week for ≥6 months prior to screening; and patient diary-confirmed criteria of ≥3 BE days per week for each of the 2 weeks prior to study baseline.

Exclusion criteria included a BMI outside the range of 18 to 45 kg/m^2^; lifetime history of bulimia nervosa or anorexia nervosa; and initiation of a formal weight loss program or psychotherapy in the 3 months prior to screening. Exclusion criteria also included a lifetime history of psychotic disorder, bipolar disorder, hypomania, or ADHD; history of moderate-to-severe depression within 6 months prior to screening; use of antidepressants, psychostimulants, or mood stabilizers within 3 months prior to screening; and a history of substance abuse in the past 12 months. Individuals were also excluded who reported a history of type I or type II diabetes, or clinically significant hypertension or cardiovascular disease.

The study was approved by an institutional review board at each study site and conducted in accordance with the International Conference on Harmonization Guideline for Good Clinical Practice and the Declaration of Helsinki. Written informed consent was obtained from all patients prior to initiation of study procedures.

### Study design

This study was conducted at 50 centers in the United States, between March 31, 2017 and May 16, 2018. Following a screening period of up to 21 days, patients were randomized (1:1:1) to receive 12 weeks of double-blind, parallel-group treatment with once-daily, fixed doses of dasotraline (4 or 6 mg), or placebo. Randomization was stratified on the baseline number of BE days per week (3-4 vs. >4 per week; from review of the patient diary for the 2 weeks before the baseline visit), and was balanced using permuted blocks. Randomization was managed by a computer-based interactive voice/web response system.^30^

Dasotraline and placebo capsules were provided in blister packs that were identical in packaging, labeling, weight, and appearance. The allocation sequence was concealed from both the study patient and all study personnel. Patients randomized to dasotraline 4 mg/d received 4 mg/d for the duration of the treatment period. Those randomized to dasotraline 6 mg/d were dosed with dasotraline 4 mg/d for the first 2 weeks of the treatment period and were then increased to 6 mg/d for the remaining duration of the treatment period. Patients who were not able to tolerate the assigned dose were discontinued from the study.

Patients who completed 12 weeks of treatment were eligible to enroll in a 12-month open-label extension study. Patients who did not enter the extension study had their medication discontinued (without taper) and participated in a 3-week medication discontinuation period intended to assess potential withdrawal effects.

### Concomitant medications

Use of the following medications was permitted during the double-blind study period for insomnia: lorazepam, temazepam, eszopiclone, zaleplon, zolpidem, zolpidem-CR, or melatonin (not to be taken in combination). The following medications were prohibited: stimulants, antidepressants, anticonvulsants, and medication associated with weight gain or weight loss, or used for the treatment of overweight or obesity.

### Efficacy assessments

The primary efficacy endpoint was mean change from baseline to week 12 in number of BE days per week, defined as a day with at least one BE episode. A patient diary, completed at home to serve as a concurrent log, was used to record the number of BE episodes per day. The diary was used as source material and reviewed by a trained rater at each study visit to determine whether each recorded eating episode met BE criteria. This assessment method has notable strengths including the reduction of recall biases.^28^

Secondary efficacy endpoints at week 12 consisted of change from baseline in the BE Clinical Global Impression of Severity (BE-CGI-S) score, proportion of patients achieving 100% cessation of BE episodes in the final 4 weeks of study participation, proportion of patients with ≥75% reduction in BE episodes, change from baseline in the Yale-Brown Obsessive–Compulsive Scale Modified for BE (YBOCS-BE) total and obsession and compulsion subscale scores,^31^ change from baseline in number of BE episodes/week, change from baseline in the eating-disorder psychopathology global score and subscale scores (restraint, eating concern, shape concern, and weight concern) on the brief version of the Eating Disorder Examination Questionnaire, modified (EDE-QM),^32^ ^,^^33^ and change from baseline on the Sheehan Disability Scale (SDS) total and subscale scores (work/school, social life, and family life/home responsibilities).^34^

### Safety and tolerability assessments

Safety assessments included adverse events (AEs), serious adverse events (SAEs), laboratory and electrocardiography (ECG) assessments, vital signs, and weight. Suicidality was assessed with the Columbia–Suicide Severity Rating Scale (C–SSRS).^35^ A monitoring plan was implemented to detect any possible diversion or abuse of dasotraline, or concurrent recreational use of nonstudy drugs. The plan included monitoring of missing or lost pills, and regular urine drug screens and breath alcohol tests. The plan also included a list of sentinel events that, if present, required additional medical surveillance. For patients who did not enter the extension study, and who discontinued study medication, potential withdrawal symptoms during the 3-week follow-up period were assessed with the Cocaine Selective Severity Assessment (CSSA)^36^; the Discontinuation-Emergent Signs and Symptoms (DESS) scale^37^; the Hamilton Anxiety Rating Scale (HAM-A)^38^; and the Montgomery-Asberg Depression Rating Scale (MADRS).^39^

### Statistical analysis

The intent-to-treat (ITT) population was defined as all randomized patients who received at least one dose of study drug and had at least one postbaseline efficacy evaluation. The safety population was defined as all randomized patients who received at least one dose of study medication. Study centers with sample sizes of 18 or fewer were pooled, based on geographic proximity.

The primary efficacy measure, and secondary efficacy measures (BE-CGI-S; YBOCS-BE; number of binge episodes per week, SDS), were analyzed using a mixed model for repeated measures (MMRM) with fixed effects terms for treatment, visit (as a categorical variable), pooled center, baseline BE days category (stratification factor), number of BE days per week at baseline, and treatment-by-visit interaction. The proportion of patients with 100% cessation of BE episodes in the final 4-weeks of treatment was analyzed using a logistic regression model with treatment, baseline binge days category (stratification factor), and baseline number of BE days per week as covariates using a last-observation-carried-forward (LOCF) approach. One secondary efficacy variable (EDE-QM), where only baseline and endpoint assessments were performed, was analyzed using an analysis of covariance (ANCOVA) model with treatment, pooled center, baseline BE days per week (stratification) as factors, and baseline number of BE days per week as a covariate.

To control the overall type I error rate strongly at 5% for the primary and key secondary endpoints, a sequential testing strategy was planned and used. Following a fixed sequence closed testing procedure (see Supplementary Figure S1), testing only proceeded conditional on the statistical significance of the test(s) of prior level(s) at a two-sided 5% significance level.

Based on results from the previous flexible-dose study,^25^ we assumed mean treatment differences (vs placebo) on the primary efficacy endpoint of 0.9 and 0.8 (common SD, 1.75) for dasotraline 4 and 6 mg/d, respectively. Therefore, it was estimated that a sample size of 96 patients per treatment group would provide at least 85% conjunctive power to reject both null hypotheses. The sample size was adjusted to 160 patients per treatment group based on a projected drop-out rate of 40%.

Descriptive statistics were used for safety variables. Rank ANCOVA was used to analyze changes in cholesterol, triglycerides, and glucose levels from baseline.

## Results

### Patients and disposition

**Figure 1.**
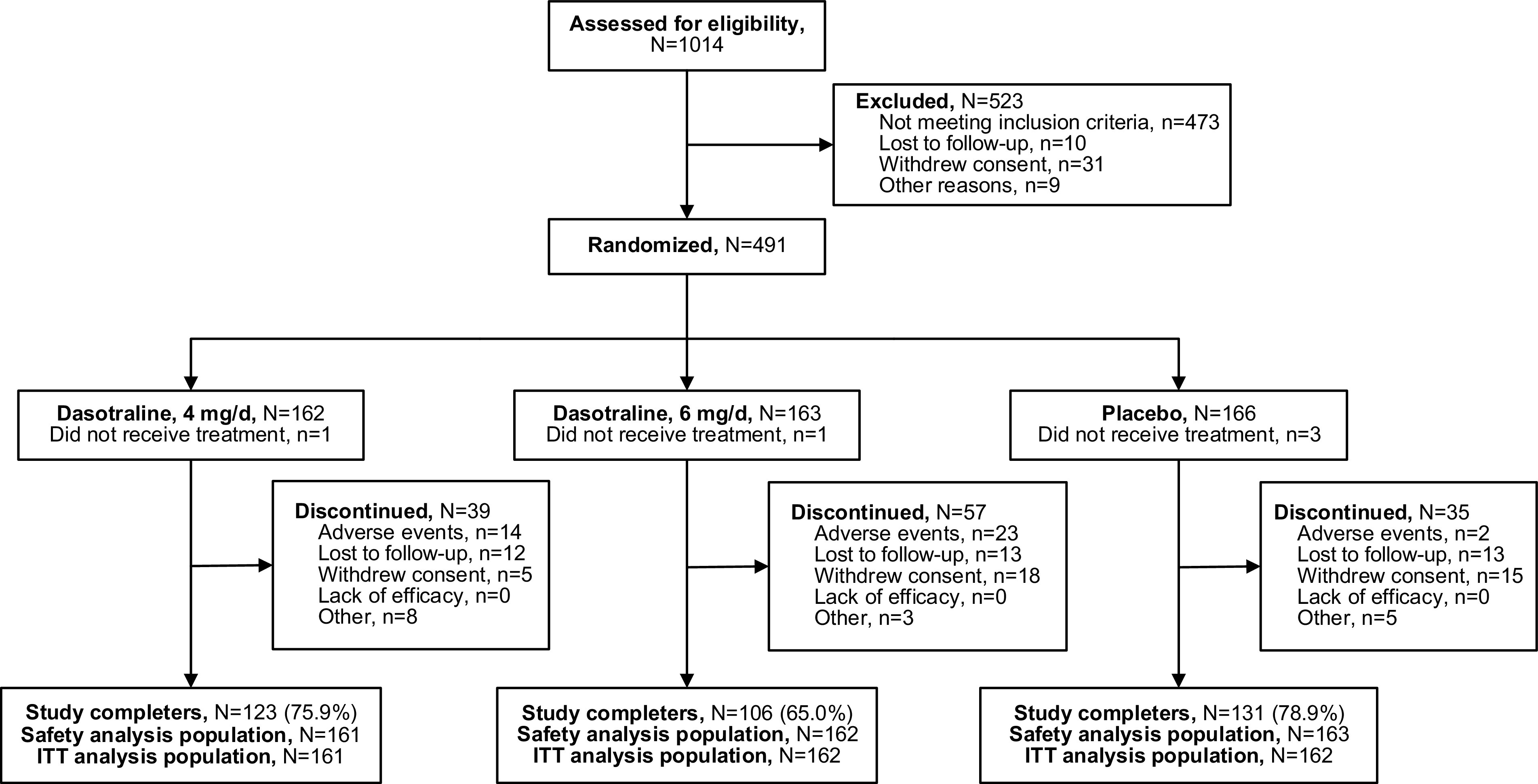
Flow diagram.

A total of 1014 patients were screened, of whom 491 were randomized to study treatment (Figure 1); 486 patients received at least one dose of study drug and 485 patients had at least one postbaseline efficacy evaluation (ITT analysis population). Twelve patients were randomized to dasotraline 6 mg/d and initially received a 4 mg dose, but never titrated up to the 6 mg dose. Safety analysis results were presented by randomized treatment group. Study completion rates were 75.9% and 65.0% for the dasotraline 4 and 6 mg dose groups, respectively, and 78.9% for placebo group; reasons for study discontinuation are summarized in Figure 1.

Clinical and demographic characteristics were comparable between treatment groups (Table 1). The overall mean age was 37.6 years; the majority of patients were white (76.3%), female (83.9%), and obese (75.5%), with a mean BMI of 34.5 kg/m^2^. The majority were not diagnosed with BED until screening despite a mean duration of nearly 13.5 years of BE symptoms. The mean baseline number of BE days per week was 4.2, and the mean number of BE episodes/wk was 5.5.

**Table 1.** Patient Demographic and Clinical Characteristics (ITT population)

	Dasotraline 4 mg/d (N = 161)	Dasotraline 6 mg/d (N = 162)	Placebo (N = 162)
Age, mean (SD), years	36.9 (9.6)	38.9 (9.7)	37.1 (10.2)
Female, n (%)	138 (85.7)	133 (82.1)	136 (84.0)
Race, n (%)			
White	119 (73.9)	121 (74.7)	130 (80.2)
Black/African American	25 (15.5)	32 (19.8)	29 (17.9)
Asian	7 (4.3)	4 (2.5)	1 (0.6)
Other	10 (6.2)	5 (3.1)	2 (1.2)
Ethnicity, n (%)			
Hispanic/Latino	31 (19.3)	26 (16.0)	28 (17.3)
Weight, kg, mean (SD)	96.9 (19.7)	96.0 (18.0)	96.9 (20.8)
BMI, kg/m^2^, mean (SD)^a^	34.8 (6.1)	34.3 (5.7)	34.5 (6.3)
Normal/Underweight (<25), n (%)	9 (5.6)	8 (4.9)	12 (7.4)
Overweight (25 to < 30), n (%)	32 (19.9)	30 (18.5)	28 (17.3)
Obesity class I (30 to <35), n (%)	41 (25.5)	48 (29.6)	42 (25.9)
Obesity class II (35 to <40), n (%)	46 (28.6)	45 (27.8)	46 (28.4)
Obesity class III (≥40), n (%)	33 (20.5)	31 (19.1)	34 (21.0)
Age, initial symptoms, mean (SD), years	23.8 (10.8)	24.1 (11.3)	24.2 (11.2)
Age, initial diagnosis, mean (SD), years	36.7 (9.7)	38.3 (10.2)	36.8 (10.4)
Binge eating days/week, mean (SD)	4.2 (1.1)	4.2 (1.1)	4.3 (1.0)
Binge eating episodes/week, mean (SD)	5.4 (3.5)	5.4 (3.0)	5.6 (2.8)
BE-CGI-S score, mean (SD)	4.5 (0.5)	4.4 (0.5)	4.4 (0.5)
YBOCS-BE total score, mean (SD)	21.6 (3.7)	21.6 (4.3)	21.9 (3.7)

Abbreviations: BMI, body mass index; BE-CGI-S, binge-eating Clinical Global Impression–Severity; ITT, intention-to-treat; SD, standard deviation; YBOCS-BE, Yale-Brown Obsessive–Compulsive Scale Modified for Binge-eating

a BMI categories based on NIH criteria.^41^

### Efficacy

**Figure 2.**
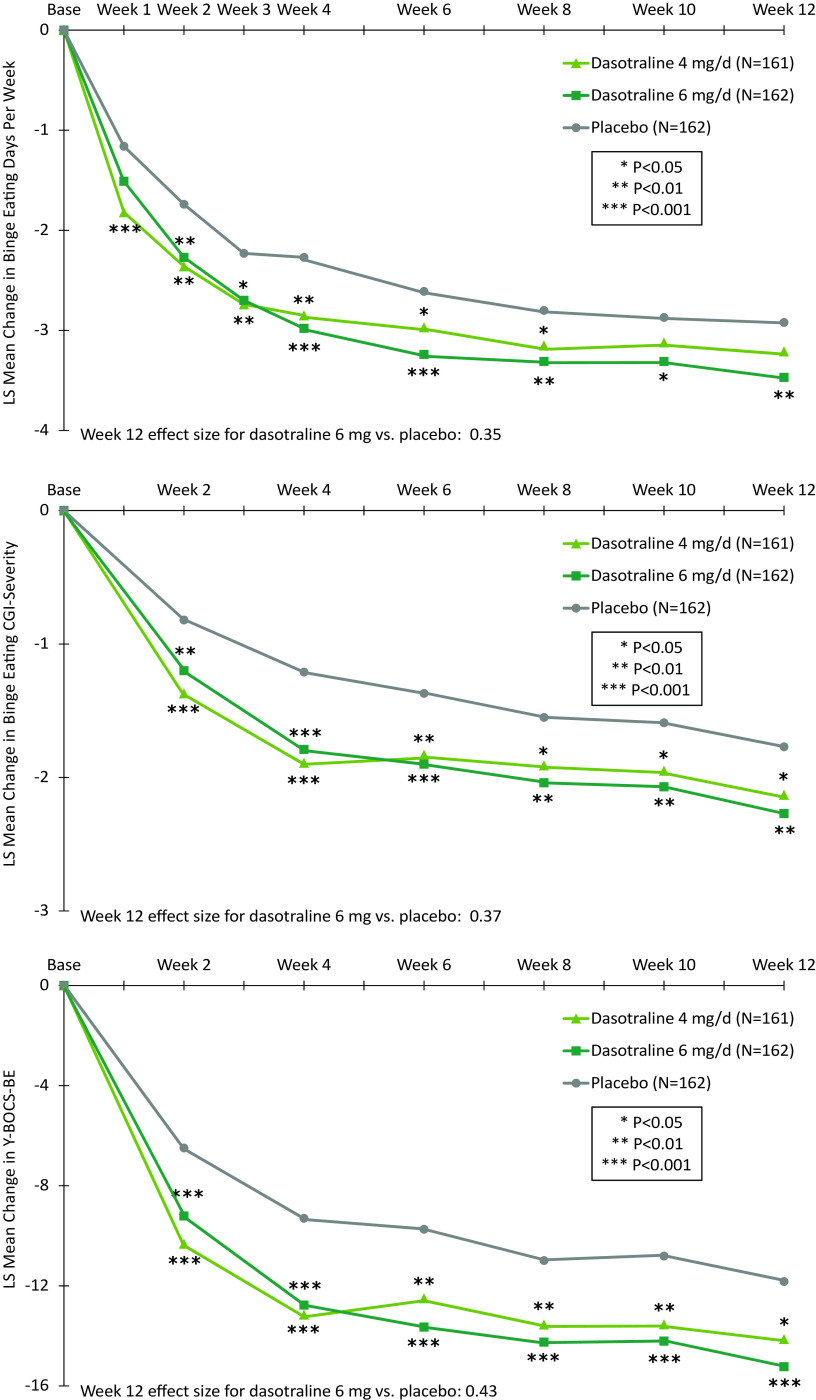
Least-squares mean change from baseline to week 12 in primary and secondary efficacy measures.

The primary analysis showed a significant reduction from baseline in the LS mean (SE) number of BE days per week for the 6 mg/d dose of dasotraline vs placebo at week 12 (−3.5 [0.1] vs −2.9 [0.1]; *P =* .0045; effect size [ES]: 0.35); treatment with the 4 mg/d dose of dasotraline did not result in a significant change vs placebo (−3.2 [0.1] vs −2.9 [0.1]; Table 2). For the 6 mg dose of dasotraline, significantly greater reduction vs placebo in BE days per week was evident at week 1 and was maintained through week 12 (Figure 2A).

**Table 2.** Primary and Secondary Efficacy Measures (ITT Population)

	Dasotraline 4 mg (N = 161)	Dasotraline 6 mg (N = 162)	Placebo (N = 162)	Treatment Difference (vs. Placebo)		
Primary efficacy variable	LS mean (SE)	LS mean (SE)	LS mean (SE)	LS mean (SE) difference (4 mg/6 mg)	Effect size (4 mg/6 mg)	*P* value^a^(4 mg/6 mg)
Binge-eating days/week	−3.2 (0.1)	−3.5 (0.1)	−2.9 (0.1)	−0.3 (0.2)/−0.6 (0.2)	0.19/0.35	.119/.0045
Secondary efficacy variables	LS mean (SE)	LS mean (SE)	LS mean (SE)	LS mean (SE) difference (4 mg/6 mg)	Effect size (4 mg/6 mg)	*P* value (4 mg/6 mg)
BE-CGI-Severity	−2.1 (0.1)	−2.3 (0.1)	−1.8 (0.1)	−0.4 (0.2)/−0.5 (0.2)	0.27/0.37	.0256/.0025
YBOCS-BE total score	−14.1 (0.7)	−15.2 (0.7)	−11.8 (0.7)	−2.3 (1.0)/−3.4 (1.0)	0.29/0.43	.0154/.0005
Obsession subscale score	−6.7 (0.3)	−7.4 (0.4)	−5.6 (0.3)	−1.2 (0.5)/−1.9 (0.5)	0.29/0.46	.0151/.0002
Compulsion subscale score	−7.3 (0.4)	−7.8 (0.4)	−6.2 (0.4)	−1.1 (0.5)/−1.5 (0.5)	0.26/0.37	.0273/.0027
EDE-QM global score (restraint, eating concern,shape concern, and weight concern)^b^	−1.20 (0.12)	−1.35 (0.12)	−0.54 (0.12)	−0.66 (0.16)/−0.81 (0.16)	0.49/0.59	<.0001/<.0001
SDS total score	−8.6 (0.55)	−8.2 (0.58)	−6.1 (0.55)	−2.4 (0.8)/−2.1 (0.8)	0.41/0.34	.0018/.0103
Work/school subscale score	−1.9 (0.19)	−1.8 (0.20)	−1.3 (0.19)	−0.6 (0.3)/−0.5 (0.3)	0.30/0.25	.0225/.0643
Social life subscale score	−3.5 (0.20)	−3.4 (0.21)	−2.4 (0.19)	−1.1 (0.3)/−1.0 (0.3)	0.50/0.44	<.0001/.0005
Family life/home subscale score	−2.9 (0.18)	−3.0 (0.19)	−2.4 (0.18)	−0.6 (0.25)/−0.6 (0.26)	0.27/0.30	.0256/.0185
Binge-eating episodes/week	−4.3 (0.2)	−4.4 (0.2)	−3.7 (0.2)	−0.6 (0.3)/−0.7 (0.3)	0.23/0.25	.0495/.0372
	**N (%)**	**N (%)**	**N (%)**	**NNT (4 mg/6 mg)**	**Odds ratio**	***P* value**
4-week BE-cessation at EOT (ITT)^c^	54/161 (33.5)	55/162 (34.0)	49/162 (30.2)	31/27	1.2/1.2	ns/ns
4-week BE-cessation at EOT^d^ (completer)	47/123 (38.2)	51/106 (48.1)	45/131 (34.4)	26/8	1.2/1.8	ns/.0298

Abbreviations: BE-CGI-S, Binge-Eating Clinical Global Impression–Severity; CI, confidence interval; EDE-QM: Eating Disorder Examination Questionnaire, modified; EOT, end of treatment; ITT, intention-to-treat; LS, least squares; ns, not significant (*P* > .05); SDS, Sheehan Disability Scale; SE, standard error; YBOCS-BE, Yale-Brown Obsessive–Compulsive Scale Modified for binge-eating.

a *P* values for the primary efficacy variable is based on hierarchical testing controlled for overall type I error; *P* values for secondary efficacy variables are nominal (exploratory) *P* values.

b Evaluated using Analysis of Covariance (ANCOVA at LOCF-endpoint).

c Proportion of patients having no binge-eating episodes in the final four study weeks was evaluated using a logistic regression model for LOCF-endpoint sample.

d This completer analysis was post-hoc.

Sensitivity analyses of the primary efficacy endpoint were performed consisting of pattern mixture models (ie, placebo-based multiple imputations and tipping point analyses per multiple imputations with penalties), permutation test, and generalized linear mixed model (GLMM) analysis. The results of these analyses supported the MMRM analysis results of the primary BE days per week outcome.

#### Secondary efficacy measures

Treatment with dasotraline 4 and 6 mg/d, respectively, was associated with greater reduction at week 12 in the BE-CGI-S score (with ES of 0.27 and 0.37), and in the YBOCS-BE total score (with ES of 0.29 and 0.43; Table 2). For the BE-CGI-S and YBOCS-BE total score, treatment group differences were evident for both dasotraline 4 and 6 mg/d at week 2, and at all subsequent assessment visits (Figure 2B,C). Treatment with dasotraline 4 and 6 mg/d, respectively, was associated with greater week 12 reduction compared to placebo in the YBOCS-BE obsession subscale (with ES of 0.29 and 0.46) and in the compulsion subscale (with ES of 0.26 and 0.37; Table 2).

The proportion of patients achieving cessation of BE episodes in the final 4 weeks of treatment was not significantly higher for either dose of dasotraline compared with placebo on the LOCF-endpoint analysis (Table 2); in a post-hoc analysis of 12-week completers, the proportion of patients achieving cessation of BE episodes in the final 4 weeks of treatment was higher for the 6 mg/d dose of dasotraline (*P* = .03; Table 2).

On additional secondary efficacy measures, treatment with dasotraline 4 and 6 mg/d, respectively, was associated with greater reduction at week 12 in BE episodes/wk (with ES of 0.23 and 0.25; Table 2). A higher proportion of patients at week 12 showed ≥75% reduction from baseline in BE episodes/wk (69.6% vs 56.2%; nominal *P* < .011; 75.9% vs 56.2%; nominal *P* = .0002).

Treatment with dasotraline 4 and 6 mg/d, respectively, was associated with greater reduction at week 12 in the SDS total score, with ES of 0.41 and 0.34; Table 2). Treatment with dasotraline 4 and 6 mg/d, respectively, was associated with greater reduction at week 12 vs placebo in the all three subscale scores, with ES ranging from 0.27 to 0.50 and 0.25 to 0.44; Table 2). Treatment with dasotraline 4 and 6 mg/d, respectively, was also associated with greater reduction at week 12 in the EDE-QM global score, with ES of 0.49 and 0.59; Table 2). Treatment with dasotraline 4 and 6 mg/d, respectively, was associated with greater LOCF-endpoint reduction at week 12 vs placebo in the all three EDE-QM subscale scores, with ES ranging from 0.36 to 0.50 and 0.44 to 0.55, respectively (Table 2).

#### Subgroup analysis of primary endpoint

In preplanned analyses, no significant treatment-by-subgroup interaction effects were observed in LS mean change at week 12 in BE days per week for gender, race, age group, ethnicity, or baseline severity (≤7 vs >7 BE episodes/wk) for either dasotraline treatment group, and for baseline BMI category for the dasotraline 4 mg/d group. A treatment interaction with respect to baseline BMI category was observed for the dasotraline 6 mg/d group (*P* = .010). The nature of the significant interaction (quantitative vs qualitative) was further evaluated using the Gail and Simon test, which indicated that the interaction was not qualitative (*P* = .875).

A post-hoc analysis was performed to examine the potential impact of body weight on treatment outcome. When patients with class III obesity (BMI ≥40 kg/m^2^) were excluded, the effect of dasotraline 4 mg/d on endpoint change in BE days per week was significant for patients in the lower weight groups (*P* = .037; ES: 0.28). In contrast, the 6 mg/d dose of dasotraline showed consistent treatment effect sizes across all baseline BMI categories.

### Safety

The most frequent treatment-emergent adverse events (≥10%) in the combined dasotraline treatment groups were insomnia, dry mouth, headache, decreased appetite, nausea, and anxiety (Table 3). The proportion of adverse events rated as severe on placebo, dasotraline 4 and 6 mg/d was 2.5%, 5.0%, and 9.9%, respectively; and the proportion discontinuing due to an adverse event was 1.2%, 8.6%, and 14.1%, respectively, with discontinuations due to insomnia occurring in 0%, 2.5%, and 2.5%, respectively.

**Table 3.** Adverse Events and Endpoint Change in Weight, BMI, Metabolic Laboratory Values and Vital Signs (Safety Population)

Treatment-emergent adverse events, n (%)^a^	Dasotraline		Placebo
	4 mg/d (N = 161)	6 mg/d (N = 162)	N = 163
Patients with any adverse event	136 (84.5)	130 (80.2)	109 (66.9)
Insomnia^b^	48 (29.8)	65 (40.1)	22 (13.5)
Dry mouth	34 (21.1)	43 (26.5)	11 (6.7)
Headache	20 (12.4)	27 (16.7)	17 (10.4)
Decreased appetite	15 (9.3)	26 (16.0)	11 (6.7)
Nausea	19 (11.8)	21 (13.0)	9 (5.5)
Anxiety	14 (8.7)	22 (13.6)	4 (2.5)
Weight decreased	14 (8.7)	12 (7.4)	2 (1.2)
Constipation	6 (3.7)	11 (6.8)	3 (1.8)
Dizziness	8 (5.0)	9 (5.6)	3 (1.8)
**Weight/BMI**	**LS Mean (SE)**	**LS Mean (SE)**	**LS Mean (SE)**
Week 12 change in weight, kg	−3.3 (0.4)*	−4.0 (0.4)*	+0.2 (0.4)
Week 12 change in BMI, kg/m^2^	−1.2 (0.1)*	−1.5 (0.1)*	+0.1 (0.1)
***Week 12 LOCF-endpoint shift***	**%**	**%**	**%**
Patients with weight reduction of ≥5% and ≥7%	30.4/18.6	31.5/18.5	4.9/1.9
Patients shifting to a higher BMI category	3.1	1.2	9.2
***Week 12 LOCF-endpoint change in lab values, mg/dL***	**Median**	**Median**	**Median**
Triglycerides	−6.0	−7.0	+1.0
Total cholesterol	−5.0	−9.5	−6.0
LDL cholesterol	−3.5	−5.0	−1.5
HDL cholesterol	−1.0	−2.0	−3.0
Glucose	1.0	1.0	0.0
Hemoglobin A1c (%)	0.0	0.0	0.0
***Week 12 LOCF-endpoint change in systolic/diastolic BP, mm Hg***	**Mean**	**Mean**	**Mean**
Standing	−0.5/+1.5	+1.3/+1.9	−1.4/−0.3
Supine	+0.3/+1.7	+1.2/+2.1	−0.5/+0.2
Orthostatic	−0.8/−0.3	+0.1/−0.2	−0.9/−0.5
***Week 12 endpoint change in pulse rate, beats/min***	**Mean**	**Mean**	**Mean**
Standing	+5.0	+7.7	+1.4
Supine	+4.8	+6.2	+0.2
Orthostatic	+0.2	+1.5	+1.1

Abbreviations: BMI, body mass index; BP: blood pressure; HDL, high-density lipoprotein; LDL, low-density lipoprotein.

a Indicates any TEAEs with a reported frequency of at least 5% in any group and the incidence in at least one dasotraline treatment group is higher than placebo.

b Combined insomnia (early, middle, and late).

* *P* < .0001 (MMRM analysis).

There were three SAEs in the dasotraline 4 mg/d group (hiatal hernia, inguinal hernia, and palpitations); one serious event in the dasotraline 6 mg/d group (psychotic disorder in patient treated previously with antipsychotics for schizoaffective disorder); and one serious event in the placebo group (cholecystitis). There were no deaths in the study.

Five patients in the dasotraline 6 mg/d group reported psychosis-related events: one each with hallucinations (moderate severity; resolved on treatment; and continued in study), paranoia (severe; resolved on treatment; and continued in study), formication (mild; not resolved; and continued in study), psychotic disorder (severe; categorized as an SAE [as noted above]; not resolved; and discontinued study), and substance-induced psychotic disorder due to use of an illicit drug (moderate; resolved; and discontinued study). No psychosis-related events occurred in the dasotraline 4 mg/d or placebo groups, and no mania-related events occurred in any treatment group.

In the subgroup of patients who completed the study and discontinued study medication (dasotraline 4 mg/d, N = 41; dasotraline 6 mg/d, N = 36; and placebo, N = 36), no signs or symptoms of withdrawal were identified based on increased severity scores on the CSSA, DESS, MADRS, or HAM-A during the 3-week discontinuation period. No patients in either treatment group had suspected or known abuse or diversion of study drug. One patient in the placebo group had a suspected abuse of alcohol, illicit substances, over-the-counter drugs, or prescription drugs obtained outside the study protocol.

Treatment with both doses of dasotraline were associated with a greater mean reduction in weight and BMI compared to placebo, with a notably high proportion of patients with a weight reduction ≥5% and ≥7% (Table 3).

There were no clinically meaningful changes in laboratory parameters in either the dasotraline 4 or 6 mg/d dose groups, except small reductions in lipids (Table 3). There were no between treatment group differences in blood pressure, heart rate, or ECG parameters.

## Discussion

In this double-blind, placebo-controlled, fixed-dose, 12-week study, involving patients with moderate-to-severe BED, dasotraline 6 mg/d significantly improved BE days per week compared to placebo treatment (*P* = .0045; primary outcome); significant reduction in BE days per week was not observed for dasotraline 4 mg/d. Dasotraline 6 and 4 mg/d treatment was associated with improvement in BE episodes per week (ES: 0.25 and 0.23, respectively) and in the BE-CGI-S, the global measure of BED severity (ES: 0.37 and 0.27, respectively).

Dasotraline 6 and 4 mg/d treatment demonstrated clinically meaningful improvement compared with placebo in the YBOCS-BE total score (ES: 0.43 and 0.29, respectively). Dasotraline treatment improved obsessional thoughts related to BE and ruminative preoccupations that interfered with daily functioning (obsession subscale), and reduced the compulsion to binge eat, increasing patient control and ability to resist the binging urges (compulsion subscale). Dasotraline 6 and 4 mg/d treatment also demonstrated clinically meaningful improvement compared with placebo in the EDE-QM scale (ES: 0.59 and 0.49, respectively), reflecting significant reductions in global eating-disorder psychopathology (cognitive features including overvaluation of shape/weight, body-image disturbance, and maladaptive restraint). Taken together, these YBOCS-BE and EDE-QM results suggest that dasotraline, even without the benefit of concomitant cognitive-behavioral therapy, provides effective treatment for core psychopathologic disturbances that represent the psychological and behavioral underpinnings of BED. Dasotraline 6 and 4 mg/d treatment improved binge-related impairment in functioning, as assessed by the patient-rated SDS (with ES of 0.34 and 0.41, respectively).

The findings of the current fixed dose study are consistent with results from a previously reported flexible dose study of dasotraline (4-8 mg/d)^25^ and provide further support for the efficacy of dasotraline in the treatment of adults with moderate-to-severe BED. As is common in fixed dose studies for psychiatric indications (including BED),^15^ ^,^^40^ smaller effect sizes were observed for dasotraline in the current study compared with the previous flexible dose study.^25^ It is also possible that the larger placebo response observed in the current fixed-dose study, when compared with the previous dasotraline and other flexible dose studies in this patient population, may have contributed to the smaller effect sizes reported here.

The lifetime comorbid substance abuse/dependence rate in patients with BED is approximately 20% to 25%.^1^ In the previous flexible dose study in BED, no evidence of abuse, misuse, or diversion of dasotraline was noted.^25^ In the current study, the lack of diversion or abuse of dasotraline, and the lack of withdrawal symptoms (as assessed by increased symptom severity scores on the CSSA, DESS, MADRS, or HAM-A), is consistent with these previous findings. In a prior double-blind, placebo and methylphenidate-comparator controlled human abuse liability study in recreational stimulant users, dasotraline doses of 8 and 16 mg were indistinguishable from placebo across all pharmacodynamic measures associated with abuse potential.^41^ Taken together, these results suggest that dasotraline may be associated with low abuse liability.

Dasotraline 4 and 6 mg/d was generally safe and well tolerated in this sample of BED patients. Despite use of a fixed dose design that did not permit dose adjustment, a relatively low percentage of adverse events were rated as severe (6.4% and 8.7%, respectively) or resulted in discontinuation (8.7%, and 14.2%, respectively). The most frequent treatment-emergent adverse events (≥10%) in the combined dasotraline treatment groups were insomnia, dry mouth, headache, decreased appetite, nausea, and anxiety. Insomnia was the most frequent adverse event and appeared to be dose-related, with rates of 29.8% and 40.1% on dasotraline 4 and 6 mg/d, respectively. However, discontinuations due to insomnia were low (2.5% for each dose).

Psychosis-related events (eg, hallucinations and paranoia) occurred in five patients in the dasotraline 6 mg/d group. One patient who experienced an event in association with use of an illicit drug, and one patient who was hospitalized for recurrence of psychosis reported previous treatment with antipsychotics for schizoaffective disorder. The remaining three psychosis-related events resolved, and the patients continued assigned treatment in the study. No psychosis-related events occurred in the dasotraline 4 mg/d or placebo groups, and no mania-related events or suicidal behavior occurred in any treatment group.

Changes in pulse and blood pressure (supine and standing) were generally small (pulse increase <10 bpm; blood pressure increase <3 mm Hg) and not clinically meaningful. No clinically meaningful effects were observed on the QTc interval or other ECG parameters.

Epidemiologic studies have reported obesity rates of 35% to 40% in individuals with a diagnosis of BED in the community.^2^ A total of 75% of patients in the current sample met NIH criteria for obesity based on BMI criteria^42^ with 20% meeting class III criteria (BMI ≥40 kg/m^2^). Treatment with dasotraline (4 and 6 mg/d, combined) was associated with clinically meaningful reduction in weight (≥5%) in 31% of patients in the total safety sample (vs 4.9% on placebo), and in 28.3% of obese patients (classes I-III) on dasotraline (vs 5.7% on placebo). Small but consistent reductions were also observed on both doses of dasotraline in metabolic parameters. Long-term studies of dasotraline are needed to determine the degree to which reduction in weight is maintained over time.

Potential limitations of the current study include exclusion of patients with clinically significant psychiatric or medical comorbidity. We note the potential limitation inherent in reliance on patient reports of BE behaviors. We emphasize, however, that our assessment method utilized patient-reported diaries as source material and that the BE criteria and frequency were determined by trained evaluators at each study visit. This assessment method has notable strengths including the reduction of recall biases^28^ and has been used previously in several trials as an endpoint.^15^ ^,^^17^ ^,^^43^ Long-term studies of dasotraline are needed to determine the degree to which the improvements are maintained over time.

## Conclusion

In this placebo-controlled trial, treatment with dasotraline 6 mg/d (but not 4 mg/d) was associated with significantly greater reduction in BE days per week. Treatment with the 4 and 6 mg/d doses of dasotraline was associated with improvement in measures of overall illness severity, in psychological and behavioral outcomes that constitute the core and associated psychopathology of BED, and in BED-related functional impairment. The findings of this fixed dose study confirm results of a previous flexible dose study, and indicate the potential of dasotraline as an efficacious, and generally well-tolerated treatment for individuals with moderate-to-severe BED.

## Supplementary material

For supplementary material accompanying this paper visit http://dx.doi.org/10.1017/S1092852920001406. click here to view supplementary material
