# Supplementary material for: Efficacy and safety of dasotraline in adults with binge-eating disorder: a randomized, placebo-controlled, fixed-dose clinical trial
Source: CNS Spectr. 2020 May 19;26(5):481–90. doi: 10.1017/S1092852920001406 (PMC8524666; doi:10.1017/S1092852920001406)
Supplement: Supplementary file 1 [file S1092852920001406sup001.pdf]

Supplemental Figure 1. Fixed Sequence of Hypothesis Testing for Primary and Secondary Efficacy Endpoints

H_1: Number of binge days per week,
dasotraline 6 mg/day vs placebo

α = 0.050

↓

H_2: Number of binge days per week,
dasotraline 4 mg/day vs placebo

↓

H_3: BE-CGI-S score,
dasotraline 6 mg/day vs placebo

↓

H_4: Subjects with a 4-week cessation,
dasotraline 6 mg/day vs placebo

↓

H_5: Y-BOCS-BE total score,
dasotraline 6 mg/day vs placebo

↓

H_6: BE-CGI-S score,
dasotraline 4 mg/day vs placebo

↓

H_7: Subjects with a 4-week cessation,
dasotraline 4 mg/day vs placebo

↓

H_8: Y-BOCS-BE total score,
dasotraline 4 mg/day vs placebo
